# A Personalized Patient-Centered Intervention to Empower through Physical Activity the Patient in the Dialysis Center: Study Protocol for a Pragmatic Nonrandomized Clinical Trial

**DOI:** 10.3390/mps3040083

**Published:** 2020-12-06

**Authors:** Fabio Manfredini, Nicola Lamberti, Yuri Battaglia, Sofia Straudi, Martino Belvederi Murri, Maria Donadi, Giovanni Piva, Fabio Fabbian, Pablo Jesús López-Soto, Luigi Grassi, Roberto Manfredini, Nino Basaglia, Alda Storari

**Affiliations:** 1 Department of Neuroscience and Rehabilitation, Section of Sports Sciences, University of Ferrara, Via Luigi Borsari 46, 44121 Ferrara, Italy; fabio.manfredini@unife.it (F.M.); pvignn@unife.it (G.P.); 2 Unit of Physical Medicine and Rehabilitation, University Hospital of Ferrara, Via Aldo Moro 8, 44124 Ferrara, Italy; sofia.straudi@unife.it (S.S.); nino.basaglia@unife.it (N.B.); 3 Unit of Nephrology and Dialysis, University Hospital of Ferrara, Via Aldo Moro 8, 44124 Ferrara, Italy; yuri.battaglia@ospfe.it (Y.B.); marydnd96@gmail.com (M.D.); a.storari@ospfe.it (A.S.); 4 Institute of Psychiatry, Department of Neuroscience and Rehabilitation, University of Ferrara, Via Fossato di Mortara 64, 44121 Ferrara, Italy; martino.belvederimurri@unife.it (M.B.M.); luigi.grassi@unife.it (L.G.); 5 Department of Medical Sciences, University of Ferrara, Via Aldo Moro 8, 44124 Ferrara, Italy; fabio.fabbian@unife.it (F.F.); roberto.manfredini@unife.it (R.M.); 6 Department of Nursing, Maimonides Biomedical Research Institute of Cordoba (IMIBIC), University of Cordoba, Reina Sofía University Hospital, 14004 Cordoba, Spain; pablo.lopez@imibic.org

**Keywords:** exercise, chronic kidney disease, exercise testing, rehabilitation, risk factors, depression

## Abstract

Sedentariness of patients affected by end-stage kidney disease (ESKD) expose them to high risk of unfavorable clinical outcomes. Exercise training is effective in improving physical function, quality of life (QoL) and long-term outcomes. However, the existing barriers related to patients, programs and dialysis staff limit patient participation and call for new strategies. This pragmatic nonrandomized trial will test the impact on ESKD population of an intervention proposed by an exercise facilitator regularly present in a dialysis center. The patient will be free to choose among three-month walking and/or resistance low-intensity training programs: (a) guided physical activity increase; (b) home-based exercise; (c) in-hospital (pre/post dialysis) supervised exercise; (d) performance assessment only. The first phase will define feasibility and the characteristics and preference of responders. The second phase will evaluate safety and patients’ adherence. Outcome measures will be collected at baseline, after three-month and at six-month follow-up. They will include: aerobic capacity, QoL, gait speed, strength, depression and long-term clinical outcomes (hospitalization and mortality). The trial was approved by the Area-Vasta Emilia-Romagna Centro Ethics Committee with approval number 48/2019. Written informed consent will be obtained from all participants. The results of the study will be presented in international congresses, published in peer-reviewed journals and communicated to the patient community. Registration details: Clinicaltrials.gov NCT04282616 [Registered:24/02/2020].

## 1. Introduction

Chronic kidney disease affects 8–16% of the population worldwide [1]. This population tends to have a progressive decrease in physical activity, cardiorespiratory fitness and muscle mass due to the combined effects of uremia and physical inactivity [2]. Low physical activity levels are associated with the worst clinical outcomes in terms of both mortality and hospitalizations, especially in end-stage kidney disease (ESKD) [3,4,5]. In addition, impaired physical performance is related to an increased risk of falls, deconditioning, sarcopenia and all-cause mortality [6,7,8,9].

The guidelines recommend that patients with ESKD be counseled and regularly encouraged by nephrologists and dialysis staff to increase their level of physical activity [10,11]. Purposely, “intra or inter-dialytic” exercise programs or structured home-based exercise programs have been proposed [12,13,14]. Recently, those types of training programs were compared in a literature review [3], in which the authors concluded that inherent advantages and concerns were present with both approaches. Indeed, despite evidence of the beneficial effects of exercise on physical fitness, mobility, health-related quality of life (QoL) and clinical outcomes in all CKD patients [2,12,15], exercise programs of any kind are exceedingly rare in nephrology units [16,17]. Moreover, participation rates in the existing programs are low, with only 6.9% of patients meeting the recommended physical activity levels [6], therefore physical inactivity remaining a hallmark of the disease [1,3].

Several authors suggest that to address these issues, new approaches to implementing exercise in dialysis patients should be considered, particularly those that focus on simplifying exercise prescription [3,4]. In particular, the authors emphasized the failure of traditional one-size-fits-all program prescriptions due to the heterogeneity of the ESKD population [3,4] and recommended the design of individualized interventions to allow patients to select the types of activities in which they engage with more autonomy [3].

To fulfill these requirements in the ESKD population, several physical, psychological and environmental barriers that are still present need to be overcome. First, the identification of sustainable programs with good adherence is a priority because of these barriers [18,19,20]. Moreover, a proactive dialysis staff attitude is another crucial issue [21,22]; unfortunately, physical activity and exercise management are not routinely addressed in the care of these patients [2]. Although motivators to exercise have been reported in other diseases [23], nurse-led disease management programs in patients on dialysis have been tested [24,25] but not for promoting physical activity.

This pragmatic trial aims to test the impact of the regular presence of an exercise facilitator in a dialysis center to promote physical activity in the ESKD population. The efficacy will be verified in terms of applicability and feasibility, and the preferred and most effective exercise pathways for the patients will be identified. Secondarily, the study aims to carry out exploratory analyses to evaluate the impact of different exercise interventions on the functional and clinical parameters of the patients.

## 2. Experimental Design and Methods

### 2.1. Trial Design

This protocol is reported following the Standard Protocol Items: Recommendations for Interventional Trials (SPIRIT) guidelines [26]. The study is a pragmatic feasibility nonrandomized implementation trial that also includes a qualitative research study.

### 2.2. Study Setting

The study will take place at the two dialysis venues of the Operative Unit of Nephrology and Dialysis located in two different locations: at the University Hospital of Cona, Ferrara and at Città della Salute, Ferrara. All the interventions scheduled, as well as the outcome measure assessment, will be performed in a purposely selected space inside each dialysis center. The study follows two different phases, as described below, to be completed within 3 years.

### 2.3. Participants

The study aims to recruit ESKD patients undergoing peritoneal or hemodialysis treatment.

Inclusion criteria: males and females patients aged ≥18 years; able to walk for at least 6 m; performing dialysis for at least 3 months in stable conditions; Mini Mental Status Examination score ≥18/30 sufficient to enable patients to give informed consent.

Exclusion criteria: uncorrected anemia (hemoglobin concentration <9 g/dL); acute infectious disease (C-reactive protein > 10 mg/L), severe cardio-respiratory (e.g., unstable angina; severe heart failure identified by New York Heart Association class III-IV), musculoskeletal or neurological conditions (e.g., lower limbs major amputation) inhibiting exercise training.

As this is an implementation feasibility trial, the exclusion criteria are kept at a minimum.

During the first meeting with potential participants, the study personnel will check patients’ interest in taking part in the study; if a positive response is provided, a screening visit to verify compliance with the inclusion criteria will be executed. Patients meeting the eligibility criteria will be given a letter explaining the study, as well as the consent form, and they will be encouraged to ask any questions. Within seven days, patients will be asked to return a signed copy of the consent form; if the patients have not yet decided, they will be given adequate time to consider their participation. According to the guidelines [26], the total number of screened subjects who are ineligible (and the reasons for their ineligibility) or not willing to participate in the study (along with the reasons and the main clinical characteristics, if provided) will be tracked.

Preparticipation screening with exercise testing will not be regularly scheduled due to the low intensity of the exercise program proposed, as it was designed for special populations [27].

For those who volunteer to participate, baseline outcome measurement sessions will be scheduled within two weeks. In this timespan, patients will be provided with a wrist fitness tracker to be worn continuously for 6 days, including at night and during resting periods. The number of daily steps, number of hours of sleep or rest and heart rate will be recorded.

## 3. Procedures

### 3.1. Phase 1

In this phase, the exercise facilitator (EF) will be set up in the dialysis center. He/she will be an experienced physiotherapist and/or qualified exercise specialist who will be active in the dialysis centers for a specified number of hours per day to counsel patients concerning their worries and answer questions concerning physical activity. The EF will be properly trained by the members of the Rehabilitation Medicine Unit at the University Hospital of Ferrara on the management of frail patients by having experience with severely disabled patients with different conditions before the beginning of the study.

Stakeholder involvement: The EF, in collaboration with the team members, will prepare the informative material for the project. Team members will arrange meetings with unit personnel and coordinators of patient groups to introduce the EF figure, to explain the aims of the project and to address doubts and questions.

After the baseline outcome assessment, the EF will propose one of the four interventions to the patients by means of a specifically designed brochure, which is explained in the following section.

Qualitative outcomes will be employed in the first phase (Table 1). The rate of patients providing consent compared to the number of eligible patients, as well as the rate of patients choosing each of the training options proposed, will be calculated to determine patients’ willingness to exercise and their preferences about training. The impact of the patients’ characteristics and other variables on the choice of the program will also be investigated.

### 3.2. Phase 2

The EF will ask each enrolled patient to select an intervention and thus enter into one of the following arms.

Unstructured physical activity (U-PA)

According to each patient’s baseline physical activity level previously measured, the EF will advise patients to start or increase their spontaneous activity by counseling them on total exercise time, mode, intensity and frequency as suggested by the guidelines [27,28] (Table 2). A log-book and a wearable physical activity monitor will be provided to each patient. The instruments have to be returned in the subsequent controls, to encourage adherence and objectively measure the exercise activities.

Structured home-based low-intensity exercise (S-HB-LI)

According to each patient’s baseline physical activity level, a semipersonalized walking program will be provided. This program, derived from previous experience with ESKD patients [13,29] was also successfully tested in other fragile populations such as stroke survivors or multiple sclerosis patients [30,31,32]. The training program will be performed at home at a prescribed speed and it includes a 10-min session/day of intermittent walking (1- or 2-min work and 1-min seated rest). The speed which is converted into a walking cadence is increased weekly and it is controlled at home with the aid of a metronome (Table 2). Patients will be provided with a daily log containing the detailed exercise prescription and free lines to provide feedback on training execution and any possible related symptoms.

Structured supervised low-intensity exercise (S-SU-LI)

Patients will meet at a specified location (room or corridor) into the dialysis unit for the exercise program in groups of a maximum of four subjects. The thirty-minute training session will be scheduled on a 2- or 3-time/week pattern, and they will be performed immediately before or after the dialysis treatment or according to patients’ preferences and considering the postdialysis fatigue that affects this population [33].

Each session will include low-intensity walking exercises (similar to those for the structured home-based training) and resistance and power exercises with elastic bands and ankle/wrist weights. Each session will begin and end with a warm-up and cool-down period of stretching, respectively. The total duration of the session will be approximately 30 min. The training intensity will be set according to the patient’s baseline capacity and it will be weekly increase. The rate of perceived exertion will be always collected (Table 2).

Physical performance assessment only (PPA)

Patients choosing this option will not begin any exercise program, but they will perform the outcome measures session only. A switch to another treatment, periodically proposed by the EF, will be always possible.

**Figure 1:**
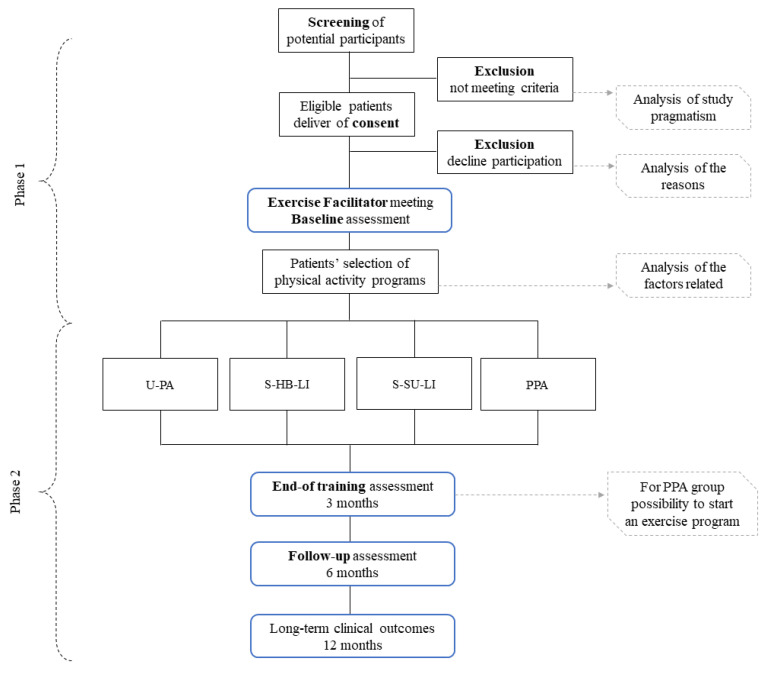
Study design. Abbreviations: U-PA-I: unstructured physical activity increase; S-HB-LI: structured home-based low-intensity; S-SU-LI: structured supervised low-intensity; PPA: physical performance assessment.

Study design is presented in Figure 1.

### 3.3. Discontinuing the Intervention

Patients who chose one of the three training options will be interviewed weekly by the EF to monitor their adherence to the program. Additionally, daily logs will be reviewed when possible. In general, all the intercurrent pathologies affecting the patients in training will be noted.

As this is a nonrandomized study in which patients will choose their preferred option, a switch within the different training modalities will always be possible. However, team members will try to motivate patients to complete the chosen 3-month program.

### 3.4. Harms

This trial, which uses well-established and safe exercise programs [13,29,30,31,32], in combination with rigorous handling of potential harms per local hospital policy, expects to minimize all the potential risks. If an undesired effect of training or a concomitant or intercurrent disease occurs, patients will be thoroughly evaluated by the team members to determine their eligibility for continuing the program, which will otherwise be stopped. Any potential harm will be treated according to the local University Hospital policy that also encompasses insurance coverage.

### 3.5. Concomitant Care and Recommendations

During the training period, patients will be asked to maintain their habitual lifestyle. Concomitant treatments (e.g., physiotherapy sessions) will be allowed, but an appropriate analysis will be carried out when analyzing the outcomes. Finally, patients will be asked to wear the same shoes and orthosis during all the testing and training sessions.

At the end of the 3-month training period, patients will be suggested to maintain a sufficient level of physical activity. To determine the amount of exercise performed during the follow-up period, patients will be asked to compile a daily log or to wear a fitness tracker.

### 3.6. Phase 2 Outcomes

First, the feasibility and safety of the exercise programs will be assessed in terms of adverse events, falls and intercurrent problems. In addition, the adherence of each patient to the selected training option will be determined by analyzing the number of sessions completed, the total minutes of training and the percentage of patients that reached the amount of physical activity indicated by the guidelines.

Quantitative outcomes will be collected before the beginning of the training program, at the end of the training program (three months) and at the six-month follow-up.

The primary outcome will be exercise capacity, assessed through the 6-min walking test. Subjects will be instructed to walk with their habitual walking device as far as possible in 6 min. The test will be performed on a 22-m walkway. The patients will have the possibility of slow down and rest if necessary. The total distance walked, the pain-free walking distance and the perceived exertion at the end of the test (Borg Scale 1–10) will be recorded [34] (Table 1).

Secondary outcomes will include the following:

- Gait speed, assessed through the 10-m walking test.
- Lower limb strength, evaluated with the 5-time sit-to-stand test.
- Health-related QoL, measured by the Italian version of the Short Form Health Survey (SF-36).
- Fear of falling, assessed through the Short Falls Efficacy Scale.
- Estimated functional capacity, measured by the Duke Activity Status Index.
- Depression, evaluated by the Beck Depression Inventory—II and the demoralization scale.
- Heart rate, oxygen saturation and blood pressure
- Laboratory outcomes: Serum creatinine levels, estimated glomerular filtration rate, Kt/v, glycaemia, total cholesterol and a full blood count will also be determined.
- Long-term outcomes: Other clinical outcomes, such as mortality and all-cause hospitalization, will be recorded at 3, 6, 12 and 24 months from the end of the exercise program.

A detailed description of secondary outcomes is available in the Supplementary Material 1.

### 3.7. Blinding

Outcome assessors will be blinded to group allocation, and unblinding will not be permissible for these researchers. If a participant interrupts the chosen program before the 3-month scheduled limit for any reason, he/she will be invited, if possible, to carry out the outcome measurement session.

### 3.8. Sample Size

To the best of our knowledge, this is the first trial of this type exploring patient preferences about programs and comparing the outcomes for these programs. Therefore, a power calculation hypothesizing a better effect of one program compared to another could not be performed; in addition, the investigators do not assign the interventions, and they do not know a priori how many patients will select each training option.

Otherwise, we aim to recruit a sufficient number of participants to address the study’s implementation objectives. The Nephrology Unit at the University Hospital of Ferrara manages approximately 140 patients on dialysis. The entrance of new patients into dialysis service will be checked every 3 months. We will try to propose the programs to all eligible patients and hypothesize a 50% rate of acceptance, which might be progressively increased by word of mouth among patients.

According to a power calculation based on a theoretical responder rate of 50% and considering a margin of error equal to 5% and a confidence level of 90%, a total of 88 patients will be required to make the first qualitative phase of the study representative of the population.

To optimize recruitment, all members of the Nephrology Unit of the hospital will be informed about the trial during team meetings. Information on the study procedures will also be given to the CKD and ESKD support groups, which will share the study information through their newsletters and website.

The EF will be available daily in the dialysis room to encourage patient participation. Informative material will also be available in the Nephrology Unit wards.

### 3.9. Data Protection

All outcome data will be recorded on different electronic spreadsheets by two researchers, and the accuracy of the data will be checked by the research coordinators. Participant data will be kept in a password-protected personal computer and stored in a properly selected closed room. The privacy of the participants and their personal medical records will be guaranteed by treating the data according to the latest European Union General Data Protection Regulation (2016/679).

### 3.10. Statistical Analysis

In the first qualitative phase, descriptive statistics, chi-squared tests and mixed models of analysis will be employed. Regression analyses will be conducted to determine if different variables (e.g., distance from dialysis center and presence of a caregiver) might interfere with patients’ decisions. Furthermore, the physical activity level and physiological and psychological variables assessed at baseline will be considered independent variables in the regression models.

In the second phase, standard methods of analysis for clinical trials will be used. First, baseline comparisons of patient characteristics for each group will be compared for demographics and primary and secondary outcome measures. The data distribution will also be verified through a Shapiro–Wilk test. A two-way repeated measure analysis of variance will be used to compare differences in the primary and secondary outcomes. Within-group comparisons will be performed via paired Student’s t-tests or Wilcoxon tests, as appropriate. If a significantly different distribution between the baseline characteristics of the group is identified, a secondary analysis that employs multivariate modeling to adjust for these factors will be performed (e.g., linear mixed models for repeated-measures analyses with unbalanced design). Moreover, Kaplan–Meier analysis and Cox regression will be used to model incidence rates for long-term clinical outcomes (hospitalization rate and death). Finally, a post hoc power analysis will also be carried out to determine the clinical impact of the findings.

All analyses will be conducted using intention-to-treat. Missing values, though we will make any effort possible to reduce their incidence, will be treated using the multiple imputation procedure. Moreover, a per-protocol analysis to assess the stability of the study’s conclusions will also be performed. Properly gender-oriented analyses will be carried out for most of the outcomes. A *p* value of 0.05 will be considered statistically significant.

Statistical analysis will be performed with SPSS 21.0 (IBM, Armonk, NY, USA) and MedCalc statistical software (version 19.4.0 and following, MedCalc Software bvba, Ostend, Belgium).

### 3.11. Data Monitoring and Interim Analysis

As this is a single-center study, a data monitoring committee will not be required. The research coordinator will be responsible for the interim analysis (in terms of recruitment and preliminary results) and for making the final decision to stop or conclude the trial.

The final dataset of the study will be completely available in a public repository.

## 4. Ethics and Dissemination

The trial was approved by the Area-Vasta Emilia-Romagna Centro Ethics Committee with approval number 48/2019. Written informed consent will be obtained from all participants.

Upon conclusion of the study, the research coordinator will be responsible for the final dataset that will be published in a public repository and accessible to researchers.

The results of this trial will be published in peer-reviewed journals, presented to local stakeholders and policymakers and shared within patients’ associations and participants’ communities.

### 4.1. Patient and Public Involvement

This starting hypothesis of the study was developed without patient involvement but based on previous suggestions and experiences. After the first drafting of the trial, the representatives of patients’ groups were invited to several meetings with the researchers. In these consultations, the trial design was exposed to patients, who gave their opinions and finally expressed their appreciation towards the study. The final registered version of the trial reflects the collaboration between the researchers and the patients’ associations.

### 4.2. Trial Limitations

This trial presents several limitations, as it is a single-center nonrandomized study. Moreover, a typical during-dialysis treatment training option is not scheduled, despite the evidence in the literature [14,15]. However, we aim to test training solutions requiring minimal space and equipment that are implementable in most dialysis centers worldwide and not overtaxing of the personnel and the routine of a dialysis center. In addition, the study design will likely introduce a selection bias in the enrolled population, as conceivably fitter patients may opt for the walking or mixed training programs, while less fit patients may choose for the unstructured increase in physical activity; proper methods of analysis will be used to overcome the lack of randomization. These factors may limit the generalizability of the findings as well the possibly small sample size that will occur in case of limited participation of patients.

## 5. Conclusions

In conclusion, this study may add a piece of missing information for exercise trials enrolling the ESKD population in terms of patients’ willingness to participate in and preferences towards exercise training and about the feasibility, safety and effectiveness of simple low-intensity programs.

The study will allow the identification of exercise interventions with few barriers that can be incorporated into clinical practice, slow patients’ physical and QoL decline and reduce hospitalizations and negative outcomes.

## Figures and Tables

**Table 1:** Outcome measures flow diagram.

	Study Period				
	Enrollment	Allocation	Post-Allocation		Close-Out
Time Point	−15 Days (Maximum)	0 Baseline	3-Month End of Training	6-Month Follow Up	12-Month
Eligibility screening	X				
Informed consent	X				
Allocation		X			
**Interventions**					
U-PA-I		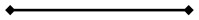			
S-HB-LI		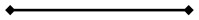			
S-SU-LI		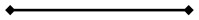			
PPA		X	X		
**Outcomes**					
*Phase 1*					
% Patients responders/eligible		X			
Physical and emotional status		X			
Exercise program choices		X			
Factors related to choices		X			
*Phase 2*					
Safety (adverse events, interruptions)			X		
Adherence (% sessions completed)			X		
6MWT (primary outcome)		X	X	X	
Gait speed, lower limbs strength, quality of life, depression, estimated capacity, fear of falling (secondary)		X	X	X	
Biochemical/laboratory markers		X	X	X	
Hospitalizations and mortality				X	X

Abbreviations: U-PA-I: unstructured physical activity increase; S-HB-LI: structured home-based low-intensity; S-SU-LI: structured supervised low-intensity; PPA: physical performance assessment; 6MWT: 6-min walk test.

**Table 2:** The different interventions proposed to the patients by the exercise facilitator.

Program	U-PA-I			S-HB-LI			S-SU-LI			PPA Performance Assessment
	Unstructured Physical Activity Increase			Structured Home-Based Low Intensity			Structured Supervised Low Intensity			
*Setting*	Home			Home			Dialysis Center			-
*Frequency*	Daily			Daily			2- or 3-Time a Week			
*Baseline 6MWD (m)*	<200	201–400	>400	<200	201–400	>400	< 200	201–400	>400	-
*Program features*	Walking (mins) Intensity: moderate [27,28]			Walking: rest (min) × reps Speed (steps/min)			Walking * + strength leg extension/leg curl/shoulder abduction (reps × series − load)			
*Week 1*	5	10	15	1:1 × 6 − 60	1:1 × 8 − 60	1:1 × 8 − 72	3 × 2 – 0.5 kg	3 × 3 − 1 kg	3 × 3 – 1.5 kg	-
*2*	5	10	15	1:1 × 6 − 63	1:1 × 8 − 63	1:1 × 8 − 76	3 × 2 – 0.5 kg	3 × 3 − 1 kg	3 × 3 – 1.5 kg	
*3*	10	15	20	1:1 × 8 − 63	1:1 × 8 − 66	1:1 × 8 − 80	4 × 2 − 1 kg	4 × 3 − 1 kg	4 × 3 – 1.5 kg	
*4*	10	15	20	1:1 × 8 − 66	1:1 × 8 − 69	1:1 × 8 − 84	4 × 2 − 1 kg	4 × 3 − 1 kg	4 × 3 – 1.5 kg	
*5*	15	15	20	1:1 × 8 − 66	1:1 × 8 − 72	1:1 × 8 − 88	3 × 3 − 1 kg	4 × 3 – 1.5 kg	4 × 3 − 2 kg	
*6*	15	20	20	1:1 × 8 − 69	1:1 × 8 − 72	1:1 × 8 − 92	3 × 3 − 1 kg	4 × 3 – 1.5 kg	4 × 3 − 2 kg	
*7*	15	20	25	1:1 × 8 − 69	1:1 × 8 − 76	2:1 × 4 − 72	4 × 3 − 1 kg	4 × 4 – 1.5 kg	4 × 4 − 2 kg	
*8*	20	20	25	1:1 × 8 − 72	1:1 × 8 − 76	2:1 × 4 − 76	4 × 3 − 1 kg	4 × 4 – 1.5 kg	4 × 4 − 2 kg	
*9*	20	20	25	1:1 × 8 − 72	1:1 × 8 − 80	2:1 × 4 − 80	4 × 3 – 1.5 kg	4 × 4 − 2 kg	4 × 4 – 2.5 kg	
*10*	20	25	25	1:1 × 8 − 76	1:1 × 8 − 80	2:1 × 4 − 84	4 × 3 – 1.5 kg	4 × 4 − 2 kg	4 × 4 – 2.5 kg	
*11*	20	25	30	1:1 × 8 − 76	1:1 × 8 − 84	2:1 × 4 − 88	4 × 4 – 1.5 kg	5 × 4 − 2 kg	5 × 4 − 3 kg	
*12*	25	25	30	1:1 × 8 − 80	1:1 × 8 − 84	2:1 × 4 − 92	4 × 4 – 1.5 kg	5 × 4 − 2 kg	5 × 4 − 3 kg	
*Monitoring*	Daily log and physical activity monitor			Daily log			Exercise facilitator			Daily log

Legend: * Walking program as S-HB-LI group; Abbreviations: 6MWD: 6-min walking distance; reps: repetitions; min: minute.

## Supplementary Materials

The following are available online at https://www.mdpi.com/2409-9279/3/4/83/s1.
