# Supplementary material for: A Personalized Patient-Centered Intervention to Empower through Physical Activity the Patient in the Dialysis Center: Study Protocol for a Pragmatic Nonrandomized Clinical Trial
Source: Methods Protoc. 2020 Dec 6;3(4):83. doi: 10.3390/mps3040083 (PMC7768449; doi:10.3390/mps3040083)
Supplement: Supplementary file 1 [file mps-03-00083-s001.pdf]

Protocol

A Personalized Patient-Centered Intervention to Empower Through Physical Activity the Patient in the Dialysis Center: Study Protocol for a Pragmatic Nonrandomized Clinical Trial

Fabio Manfredini [1,2], Nicola Lamberti [1,*], Yuri Battaglia [3], Sofia Straudi [2], Martino Belvederi Murri [4], Maria Donadi [3], Giovanni Piva [1], Fabio Fabbian [5], Pablo Jesús López-Soto [6], Luigi Grassi [4], Roberto Manfredini [5], Nino Basaglia [2] and Alda Storari [3]

[1] Department of Neuroscience and Rehabilitation, Section of Sports Sciences, University of Ferrara, Via Luigi Borsari 46, 44121 Ferrara, Italy; fabio.manfredini@unife.it (F.M.); pvignn@unife.it (G.P.)
[2] Unit of Physical Medicine and Rehabilitation, University Hospital of Ferrara, Via Aldo Moro 8, 44124 Ferrara, Italy; sofia.straudi@unife.it (S.S.); nino.basaglia@unife.it (N.B.)
[3] Unit of Nephrology and Dialysis, University Hospital of Ferrara, Via Aldo Moro 8, 44124 Ferrara, Italy; yuri.battaglia@ospfe.it (Y.B.); marydnd96@gmail.com (M.D.); a.storari@ospfe.it (A.S.)
[4] Institute of Psychiatry, Department of Neuroscience and rehabilitation, University of Ferrara, Via Fossato di Mortara 64, 44121 Ferrara, Italy; martino.belvederimurri@unife.it (M.B.M.); luigi.grassi@unife.it (L.G.)
[5] Department of Medical Sciences, University of Ferrara, Via Aldo Moro 8, 44124 Ferrara, Italy; fabio.fabbian@unife.it (F.F.); roberto.manfredini@unife.it (R.M.)
[6] Department of Nursing, Maimonides Biomedical Research Institute of Cordoba (IMIBIC), University of Cordoba, Reina Sofía University Hospital, 14004 Cordoba, Spain; pablo.lopez@imibic.org
* Correspondence: nicola.lamberti@unife.it; Tel.: +39-053-223-6187

Received: 11 November 2020; Accepted: 3 December 2020; Published: date

Secondary outcomes

Secondary outcomes will include:

- Gait speed, assessed through the 10-meter walking test [A1]. Patients will be positioned at the start of a 10-meter corridor marked with four lines, respectively at 0, 2, 8 and 10 meters from the start. Patients will be asked to walk as fast as they can, but safely, from the first to the last line, using their habitual walking device. A skilled operator will record the time within the two middle lines, that will be converted into speed. Two trails are allowed for this test, and the mean of the two results will be considered
- Lower limb strength, evaluated with the 5-time sit-to-stand test. This test, widely employed in CKD patients [A2,15], consists in moving from a sitting position to a standing position on a 42-cm high chair as quick as possible, for five times, with arms folded across the chest. Time will be taken when the test starts, and when the patient lay his/her buttocks on the chair for the fifth time. For patients unable to complete any repetition, an arbitrary time of 120 seconds will be assigned.
- Health-related quality of life (QoL), measured by the Italian version of Short Form Health Survey (SF-36) [A3]. This questionnaire consists of 36 items with a score ranging from 0 to 100 divided into 8 domains examining various aspects of health-related QoL.
- Fear of falling, assessed through the Short Falls Efficacy Scale [A4]. This scale is composed of seven 4-point Likert questions about how concerned the patient is about the possibility of falling
- Estimated functional capacity, by the Duke Activity Status Index [A5]. This is an assessment tool to evaluate the functional capacity of patients through 12 dichotomous (yes/no) questions covering various aspects of life. The total score, that ranges from 0 to 58.2 when higher scores indicate a higher functional capacity, will be calculated.

- Depression, evaluated by the Beck Depression Inventory—II [A6]. This questionnaire, composed of 21 multiple-choice questions, is suitable for measuring the gravity of clinical depression in adults and in diseased populations.
- Demoralization, assessed by the Demoralization scale [A7]. This questionnaire is a multidimensional instrument used primarily as a unidimensional measure of demoralization, and it is composed of 24 items and 5 factors.
- Laboratory outcomes: values of serum creatinine, estimated glomerular filtration rate, Kt/v, glycaemia, total cholesterol and full blood count will be also collected.
- Long-term outcomes. Other clinical outcomes as mortality and all-cause hospitalizations will be recorded at 3-, 6-, 12- and 24-month from the end of exercise program. In addition, for CKD patients, time to the eventual beginning of dialysis will also be collected.

Supplementary references

[A1] Mossberg, K.A. Reliability of a timed walk test in persons with acquired brain injury. *Am. J. Phys. Med. Rehabil.* **2003**, *82*, 385–390.

[A2] Segura-Ortí, E.; Kouidi, E.; Lisón, J.F. Effect of resistance exercise during hemodialysis on physical function and quality of life: randomized controlled trial. *Clin. Nephrol*. **2009**, *71*; 527–537.

[A3] Apolone, G.; Mosconi, P. The Italian SF-36 Health Survey: translation, validation and norming. *J. Clin. Epidemiol*. **1998**, *51*, 1025–1036.

[A4] Yardley, L.; Beyer, N.; Hauer, K.; Kempen, G.; Piot-Ziegler, C.; Todd, C.; Development and initial validation of the Falls Efficacy Scale-International (FES-I). *Age. Ageing*. **2005**, *34*, 614–619.

[A5] Hlatky, M.A.; Boineau, R.E; Higginbotham, M.B; Lee, K.L.; Mark, D.B.; Califf, R.M.; Cobb, F.R.; Pryor, D.B. A brief self-administered questionnaire to determine functional capacity (the Duke Activity Status Index). *Am. J. Cardiol*. **1989**, *64*, 651–654.

[A6] Arnau, R.C.; Meagher, M.W.; Norris, M.P.; Bramson, R. Psychometric evaluation of the Beck Depression Inventory-II with primary care medical patients. *Health. Psychol*. **2001**, *20*, 112–119.

[A7] Kissane, D.W.; Wein, S; Love, A; Lee, X.Q.; Kee, P.L.; Clarke, D.M. The Demoralization Scale: a report of its development and preliminary validation. *J. Palliat. Care*. **2004**,*20*, 269–276.
